# Preparation of ZnFe_2_O_4_/α-Fe_2_O_3_ Nanocomposites From Sulfuric Acid Leaching Liquor of Jarosite Residue and Their Application in Lithium-Ion Batteries

**DOI:** 10.3389/fchem.2018.00442

**Published:** 2018-09-25

**Authors:** Jinhuan Yao, Jing Yan, Yu Huang, Yanwei Li, Shunhua Xiao, Jianrong Xiao

**Affiliations:** Guangxi Key Laboratory of Electrochemical and Magneto-Chemical Functional Materials, College of Chemistry and Bioengineering, Guilin University of Technology, Guilin, China

**Keywords:** lithium-ion batteries, ZnFe_2_O_4_/α-Fe_2_O_3_ composites, anode materials, jarosite residue, chemical coprecipitation method

## Abstract

Recycling Zn and Fe from jarosite residue to produce high value-added products is of great importance to the healthy and sustainable development of zinc industry. In this work, we reported the preparation of ZnFe_2_O_4_/α-Fe_2_O_3_ nanocomposites from the leaching liquor of jarosite residue by a facile chemical coprecipitation method followed by heat treatment at 800°C in air. The microstructure of the as-prepared ZnFe_2_O_4_/α-Fe_2_O_3_ nanocomposites were characterized by X-ray diffraction (XRD), Mössbauer spectroscopy, scanning transmission electron microscope (STEM), and X-ray photoelectron spectrum (XPS). The results demonstrated that the ZnFe_2_O_4_/α-Fe_2_O_3_ composites are composed of interconnected ZnFe_2_O_4_ and α-Fe_2_O_3_ nanocrystals with sizes in the range of 20–40 nm. When evaluated as anode material for Li-ion batteries, the ZnFe_2_O_4_/α-Fe_2_O_3_ nanocomposites exhibits high lithium storage activity, superior cyclic stability, and good high rate capability. Cyclic voltammetry analysis reveals that surface pseudocapacitive lithium storage has a significant contribution to the total stored charge of the ZnFe_2_O_4_/α-Fe_2_O_3_, which accounts for the enhanced lithium storage performance during cycling. The synthesis of ZnFe_2_O_4_/α-Fe_2_O_3_ nanocomposites from the leaching liquor of jarosite residue and its successful application in lithium-ion batteries open up new avenues in the fields of healthy and sustainable development of industries.

## Introduction

As one of the most promising energy storage systems, rechargeable lithium-ion batteries (LIBs) have been widely applied in portable electronic devices, electric vehicles, and smart grids (Dunn et al., 2011; Scrosati et al., 2011; Larcher and Tarascon, 2014). To meet the ever-increasing requirements of high power density and high energy density, developing new electrode materials with high specific capacity and high rate capability is very crucial for manufacturing the next-generation LIBs (Tang et al., 2015; Yi et al., 2016; Zhu and Yi, 2016; Massé et al., 2017; Yao et al., 2018a). The commercial graphite anode material for LIBs suffers from low theoretical capacity (about 372 mAh g^−1^), poor rate performance, and serious safety issues, and therefore cannot fulfill demands for LIBs with high energy density, high power density, and good safety in operation (Goriparti et al., 2014; Yi et al., 2015; Zhang et al., 2018). Over recent years, transition metal oxides (TMOs) have attracted extensive attention for their high theoretical specific capacities (600–1,200 mAh g^−1^) and good safety as anode materials for LIBs (Reddy et al., 2013; Nitta and Yushin, 2014; Yuan et al., 2014; Pan et al., 2015b; Zhu et al., 2015). Among the various TMOs, Fe-based oxides have been widely studied due to their abundance, low cost, safety, wide availability, and environmental friendliness (Zhang et al., 2014; Pan et al., 2015a; Xu et al., 2015; Yao et al., 2018c; Zheng Z. M. et al., 2018). In particular, ZnFe_2_O_4_ and Fe_2_O_3_ stand out from the Fe-based oxides because of their high theoretical capacities (1,072 mAh g^−1^ for ZnFe_2_O_4_ and 1,007 mA h g^−1^ for Fe_2_O_3_). However, the poor power capability, and fast capacity fading owning to the huge volume changes during discharge/charge cycles for ZnFe_2_O_4_ and Fe_2_O_3_, severely hinders their practical applications in LIBs (Reddy et al., 2013; Zhang S. L. et al., 2017; Yao et al., 2018b). Constructing appropriate nanostructures has been demonstrated to be an effective way to improve the electrochemical performance of electrode active materials (Zhang et al., 2013; Yu et al., 2018). Various nanostructured ZnFe_2_O_4_ and Fe_2_O_3_ materials have been fabricated using diverse methods, such as sol-gel method (Thankachan et al., 2015), hydrothermal synthesis (Lin and Pan, 2015; Li L. L. et al., 2017; Zheng Z. M. et al., 2018), solvothermal synthesis (Lu et al., 2013; Yang et al., 2017), high energy ball-milling, electrospinning method (Wang C. L. et al., 2017), and reactive pulsed laser deposition method (NuLi et al., 2004). Although improved electrochemical performances have been achieved, most of the reported methods are generally time-consuming and involving complicated steps, high cost due to high energy consumption and expensive raw materials, and difficult to scale up, which greatly restrict the development and practical application of nanostructured ZnFe_2_O_4_ and Fe_2_O_3_ materials in LIBs. Therefore, it is still a big challenge but urgent demand to pursue a facile, efficient, and inexpensive method for massive production of nanostructured Fe-based oxide electrode materials with high performance for next-generation LIBs. Recycling waste materials can ease the resource crisis, reduce environmental pollution, and create new economic values, which has a great practical significance for the healthy and sustainable improvement of the human living environment. Huge quantities of jarosite residue was produced from traditional zinc hydrometallurgy process in the world. Most of the jarosite residue was stored up, which takes massive land and brings forth great risk of environmental pollution (Ju et al., 2011). The leaching liquor (containing Zn and Fe) of jarosite residue can be directly used to prepare high value-added Fe-based oxides functional materials, which has not been reported until now.

Herein, we reported the use of leaching liquor of jarosite residue to prepare ZnFe_2_O_4_/α-Fe_2_O_3_ nanocomposites by a chemical coprecipitation method followed by heat treatment at 800°C in air. This method offers the merits of low-cost, facile, and scalable production of nanostructured ZnFe_2_O_4_/α-Fe_2_O_3_ composites. When studied as anode materials for LIBs, the as-prepared porous ZnFe_2_O_4_/α-Fe_2_O_3_ nanocomposites exhibit high reversible capacity, excellent cycling stability and good high rate capability, which can be attributed to the synergetic effect between ZnFe_2_O_4_ and α-Fe_2_O_3_ nanoparticles and the significant pseudocapacitive behavior during discharging and charging processes.

## Experimental

### Preparation of ZnFe_2_O_4_/α-Fe_2_O_3_ nanocomposites

All chemicals employed in this work were of analytical reagent grade and used without further purification. The leaching liquor contains 44 mM Zn^2+^, 121 mM Fe^2+^, and a small amount of Cu^2+^, Ca^2+^, As^3+^, and In^3+^. In a typical procedure, 0.464 g ZnSO_4_·7H_2_O was dissolves in 100 mL leaching liquor so that the molar ratio of Zn^2+^ and Fe^3+^ in the solution is 1:2. After ultrasonic treatment for 10 min, an aqueous solution of NH_4_OH (200 mL, ~2.67 M) was added dropwise into the above mixed solution under constant vigorous stirring at room temperature. After continuous stirring for 3 h, the deposit was kept in the mother solution at room temperature for 12 h and allowed to settle, then washed with deionized water, filtered, and dried in an oven at 80°C overnight to get the precursor. Finally, the as-prepared precursor was calcinated at 800°C for 2 h in air to obtain the ZnFe_2_O_4_/α-Fe_2_O_3_ sample.

### Physical characterization

The crystal structure of the as-prepared sample was identified with a powder X-ray diffractometer (XRD, Dutch PANalytica X'Pert^3^ powder) with Cu Kα radiation (λ = 1.5406 Å). The X-ray tube voltage and current were set at 40 kV and 40 mA, respectively. Field-emission scanning electron microscope (FESEM, Hitachi SU500) and transmission electron microscope (HRTEM, JEM-2100F) were used to analyze the microstructure of the as-prepared sample. ^57^Fe Mössbauer spectrum was recorded by using an MFD-500A Mossbauer spectrometer (Topologic Systems, Japan). To determine the surface composition and chemical states of the sample, X-ray photoelectron spectroscopy (XPS) analysis was carried out using an ESCALAB 250 spectrometer (Perkin-Elmer), with an Al Kα source (1486.6 eV) operated at 15 kV and 150 W, at a base pressure of 2 × 10^−9^ Torr.

### Electrochemical measurements

The electrochemical performance of the ZnFe_2_O_4_/α-Fe_2_O_3_ nanocomposites was evaluated in CR2032-type coin cells assembled in an argon-filled glovebox. The test electrodes were fabricated by mixing active materials (ZnFe_2_O_4_/α-Fe_2_O_3_ nanocomposites), Super-P carbon black, and binder (polyvinylidene difluoride, PVDF) with a weight ratio of 6:3:1 in Nmethyl-2-pyrrolidone (NMP) solvent. The obtained slurry was uniformly coated onto a copper foil and then dried under at 80°C for 12 h in a vacuum oven. The mass loading of the working electrode is ~1.0 mg cm^−1^. Metallic lithium foils were used as both the counter and reference electrodes. A Celgard 2400 microporous polypropylene membrane was used as the separator. The electrolyte was 1M LiPF_6_ solution, composing of ethylene carbonate (EC), diethyl-carbonate (DEC), and dimethyl carbonate (DMC) (1:1:1, in volume). Cyclic voltammetry (CV) and electrochemical impedance spectroscopy (EIS) profiles were carried out on a CHI760E electrochemical workstation. The CVs were recorded within the potential range of 0.01–3.0 V (vs. Li/Li^+^) at various of scanning rates. The EIS spectra were measured in the frequency ranging from 100 mHz to 100 kHz by applying an AC amplitude of 5 mV at a fully charged state. The galvanostatic discharge/charge measurements were performed on a NEWARE battery testing system in the voltage range of 0.01–3.0 V (vs. Li/Li^+^). All the electrochemical tests were performed at room temperature.

## Results and discussion

### Structure characterization

**Figure 1:**
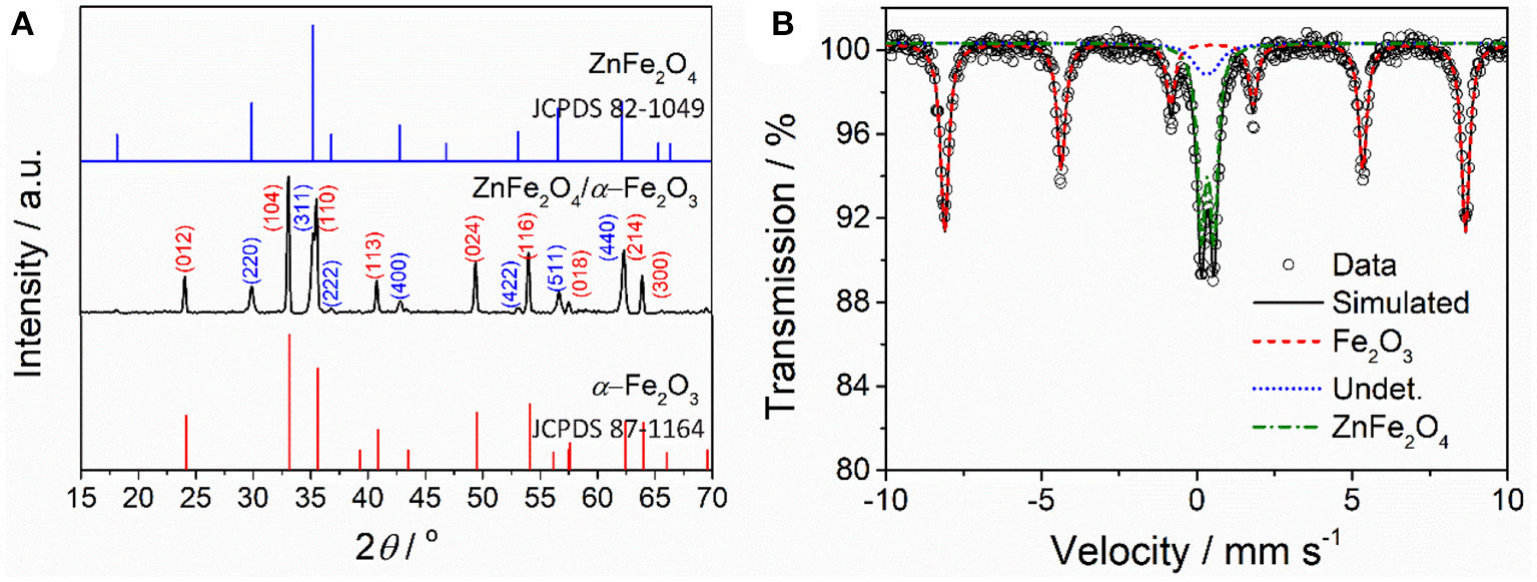
**(A)** XRD pattern and **(B)** room-temperature Mössbauer spectrum of the as-prepared ZnFe_2_O_4_/α-Fe_2_O_3_ nanocomposites.

**Figure 2:**
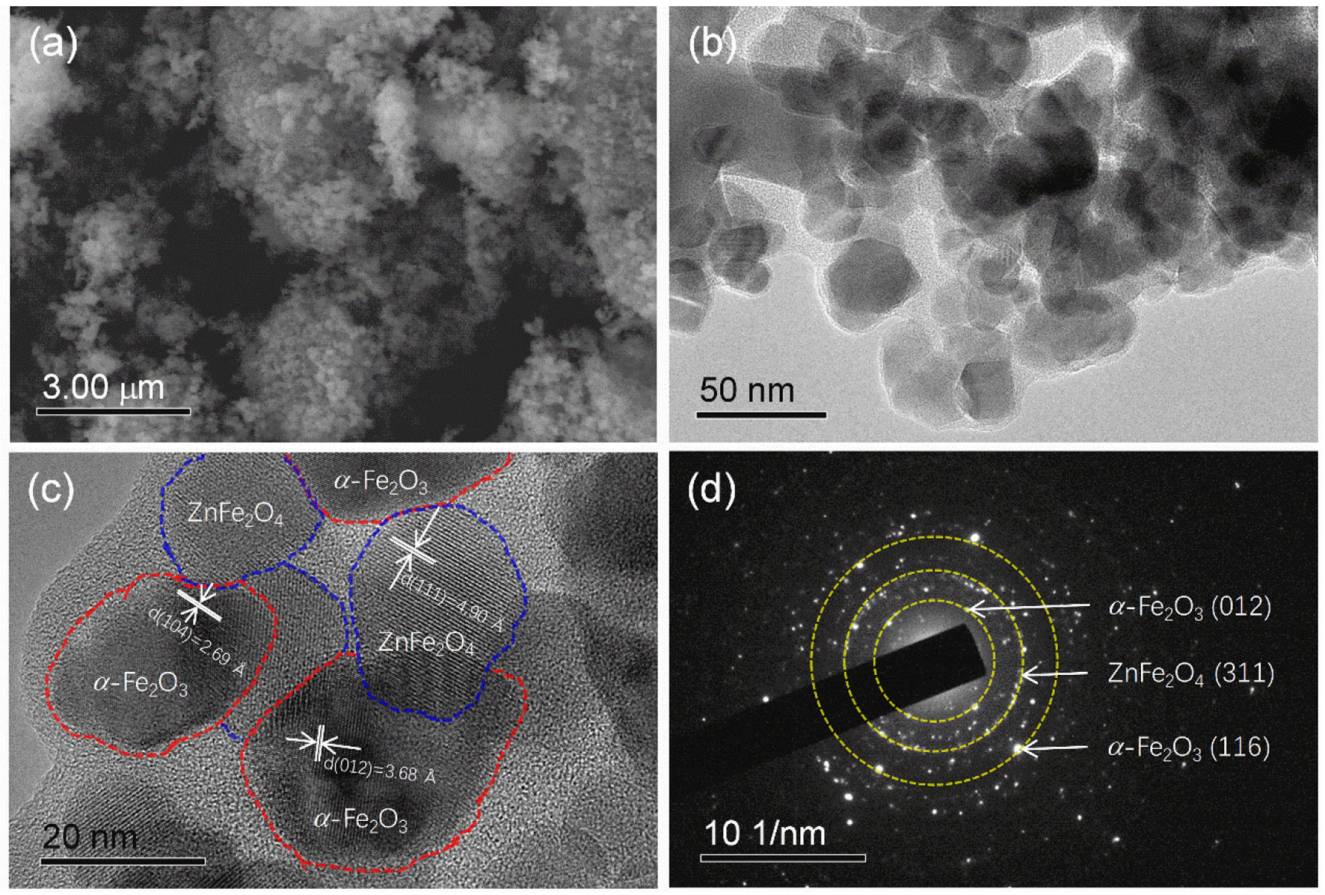
**(a)** SEM image, **(b,c)** STEM imanges, and **(d)** SAED pattern of the as-prepared ZnFe_2_O_4_/α-Fe_2_O_3_ nanocomposites.

Figure 1 shows XRD pattern of the as-prepared ZnFe_2_O_4_/α-Fe_2_O_3_ nanocomposites. The diffraction peaks of the nanocomposites can be well indexed to either rhombohedra α-Fe_2_O_3_ (JCPDS 87-1164) or spinel ZnFe_2_O_4_ (JCPDS 82-1049). No diffraction peaks from impurities are detected, suggesting the nanocomposites are composed of spinel ZnFe_2_O_4_ phase and α-Fe_2_O_3_ phase. The average crystal sizes of the ZnFe_2_O_4_ phase and α-Fe_2_O_3_ phase in the nanocomposites estimated using Scherrer's formula are about 30 and 44 nm, respectively. ^57^Fe Mössbauer spectroscopy was used to determine the phase composition and metal cation redistribution in the ZnFe_2_O_4_/α-Fe_2_O_3_ nanocomposites and the result is presented in Figure 2b. The dots in Figure 2b are experimental spectrum and the continuous curves are fitting lines. Table 1 summarizes the Mössbauer refined parameters from the fitting of the spectrum. The Mössbauer spectrum of the ZnFe_2_O_4_/α-Fe_2_O_3_ nanocomposites are fitted with one sextet and two doublets. The sextet can be assigned to α-Fe_2_O_3_ with iron content 63% in the composites (Pailhé et al., 2008; Lazarević et al., 2014), and the doublets can be due to super paramagnetic ZnFe_2_O_4_ with iron content 37% in the composites (Yao et al., 2012; Amir et al., 2018). The doublet with a lower quadrupole splitting can be assigned to the Fe^3+^ at tetrahedral sites in ZnFe_2_O_4_ (Amir et al., 2018). The site occupation in ZnFe_2_O_4_ can be represented by (Zn_1−λ_Fe_λ_)_tet_[Zn_λ_Fe_2−λ_]_oct_O_4_ where λ is the inversion parameter (0 ≤ λ ≤ 1). The calculated inversion parameter of the ZnFe_2_O_4_ phase is 0.22. Thus, the chemical formula of the ZnFe_2_O_4_ phase in the nanocomposites can be expressed as (Zn_0.78_Fe_0.22_) [Zn_0.22_Fe_1.78_]O_4_.

**Table 1:** Mössbauer parameters for the ZnFe_2_O_4_/α-Fe_2_O_3_ nanocomposites.

**Fitting lines**	**Assignment of sites**	**IS (mm s^−1^)**	**QS (mm s^−1^)**	***R*_A_ (%)**
Sextet	Fe_2_O_3_	0.376	−0.220	63.2
Doublet	Fe^3+^(A)_tet_	0.319	0.384	7.6
Doublet	Fe^3+^[B]_oct_	0.348	0.406	29.2

Figure 2a presents the SEM image of the as-prepared ZnFe_2_O_4_/α-Fe_2_O_3_ nanocomposites. It can be seen that the sample is composed of agglomerated nanosized primary particles. More detail structure information about the ZnFe_2_O_4_/α-Fe_2_O_3_ nanocomposites was obtained from STEM analyses. The low- and high-magnification STEM images (Figures 2b,c) show that the ZnFe_2_O_4_/α-Fe_2_O_3_ nanocomposites are actually composed of interconnected nanocrystals with size in the range of 20~50 nm, which is consistent with the result from the XRD analysis. The ZnFe_2_O_4_ nanocrystals and α-Fe_2_O_3_ nanocrystals could be well distinguished in the high-resolution STEM image (Figure 2c). The selected area electron diffraction (SAED, Figure 2d) pattern of the ZnFe_2_O_4_/α-Fe_2_O_3_ nanocomposites suggests its polycrystalline character and the distinct diffraction spots could be assigned sequentially to α-Fe_2_O_3_ (012), ZnFe_2_O_4_ (311), and α-Fe_2_O_3_ (116) planes from the center, which also appear in the XRD pattern (Figure 1A). The primary nanocrystals of the ZnFe_2_O_4_/α-Fe_2_O_3_ nanocomposites offer large surface area and short diffusion pathways for fast Li^+^ diffusion; the interconnected primary nanocrystals provide continuous electronic transfer channels in the ZnFe_2_O_4_/α-Fe_2_O_3_ composites; the large amount of void spaces among the interconnected nanocrystals would benefit the penetration of electrolyte in electrode, and accommodate the strain induced by the volume change upon discharge/charge cycling. Moreover, the interconnected ZnFe_2_O_4_/α-Fe_2_O_3_ heterojunctions could provide an enhanced inner electric field at the interface between ZnFe_2_O_4_ and α-Fe_2_O_3_ nanocrystals, which will greatly accelerate the charge-transfer kinetics during electrochemical reactions (Qiao et al., 2013; Zhao et al., 2014; Zheng et al., 2016).

**Figure 3:**
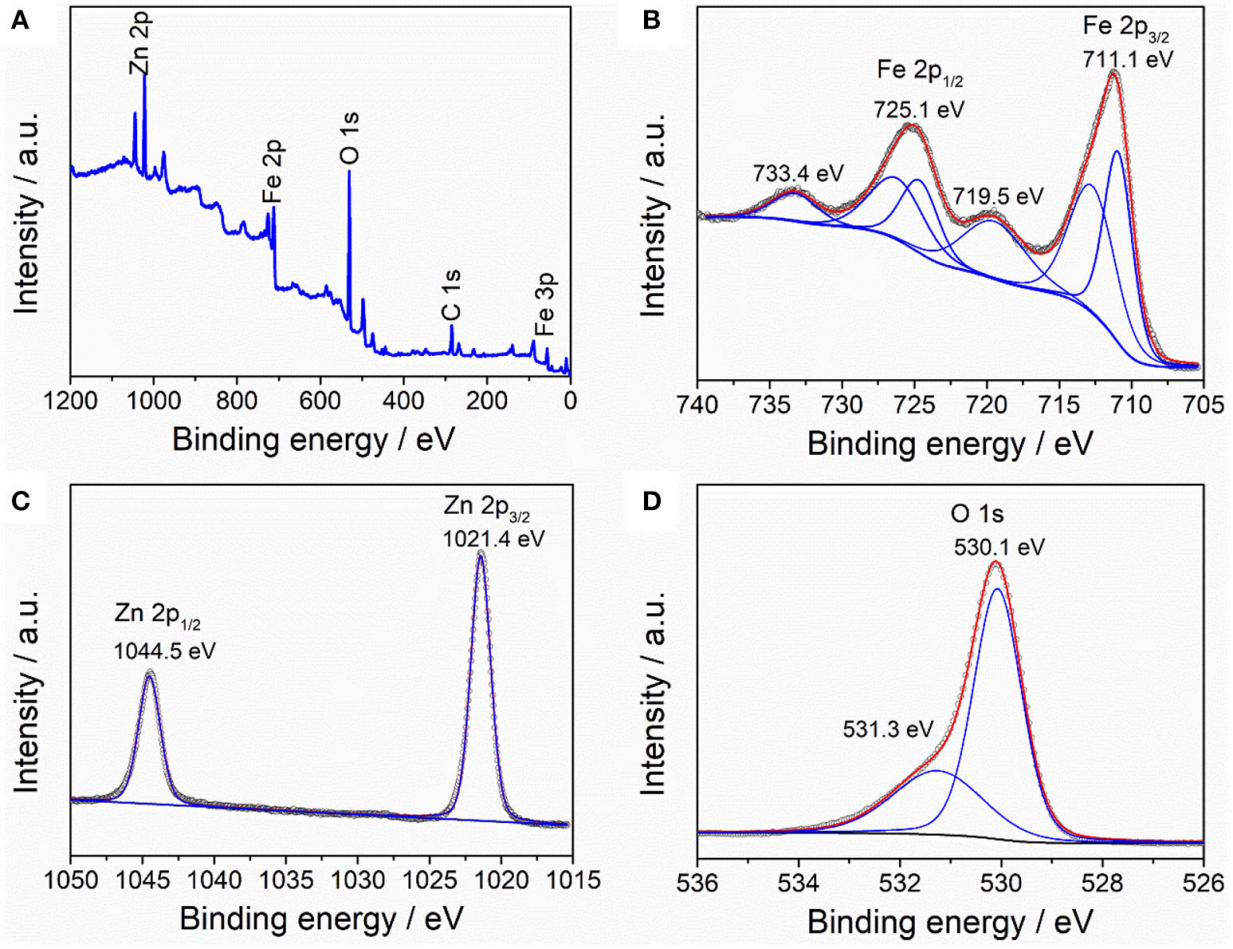
XPS spectra of ZnFe_2_O_4_/α-Fe_2_O_3_ nanocomposites: **(A)** survey of the sample, **(B)** Fe 2p, **(C)** Zn 2p, and **(D)** O 1s.

The composition and element valence of the as-prepared ZnFe_2_O_4_/α-Fe_2_O_3_ nanocomposites were characterized by XPS and the result are displayed in Figure 3. The survey XPS spectrum (Figure 3A) shows the presence of elements Zn, Fe, and O, as well as C, which comes from the adsorbed CO_2_ and/or hydrocarbon contaminations.

The Fe 2p core peak spectrum shows two main peaks at 711.1 and 725.1 eV, which can be assigned to Fe_3/2_ and Fe_1/2_, respectively (Guo et al., 2014). The presence of two satellite peaks at 719.5 and 733.4 eV is characteristic of the Fe^3+^ in the ZnFe_2_O_4_/α-Fe_2_O_3_ nanocomposites (Zhao et al., 2014). The Zn 2p core peak spectrum is composed of two intense peaks at 1021.4 and 1044.5 eV, which can be ascribed to Zn 2p_3/2_ and Zn 2p_1/2_ of Zn^2+^, respectively (Lu et al., 2017). The O 1s core peak spectrum consists of two peaks at 530.1 and 531.3 eV, which can be attributed to the lattice oxygen in the nanocomposites and surface-adsorbed hydrocarbon (Zhang L. H. et al., 2017).

### Electrochemical characterization

**Figure 4:**
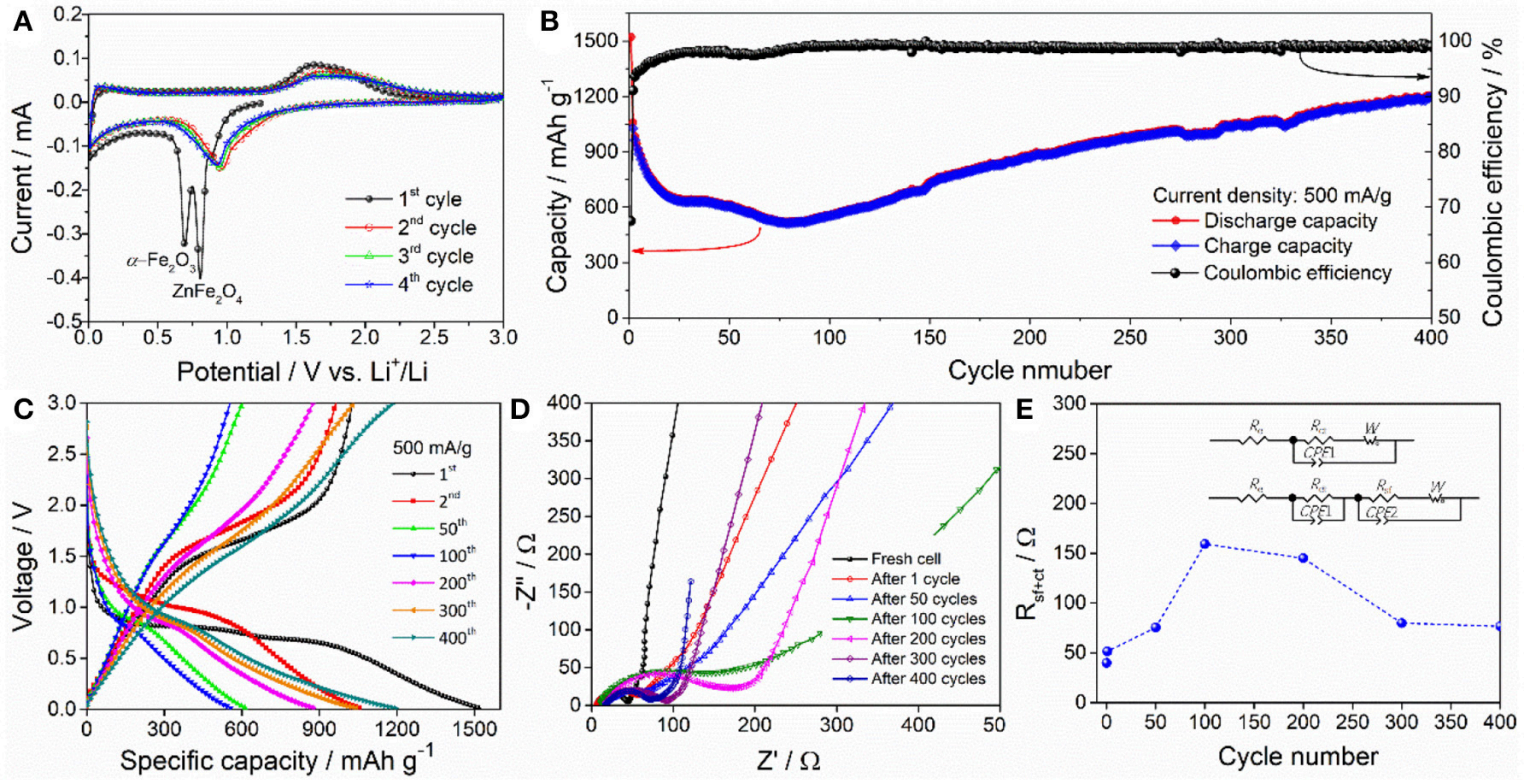
**(A)** The first four CV curves of the ZnFe_2_O_4_/α-Fe_2_O_3_ nanocomposites electrode at 0.1 mV/s. **(B)** Cycling performance and **(C)** selected discharge/charge profiles of the ZnFe_2_O_4_/α-Fe_2_O_3_ nanocomposites electrode at 500 mA/g. **(D)** Nyquist plots and **(E)** *R*_sf+ct_ values of the ZnFe_2_O_4_/α-Fe_2_O_3_ nanocomposites electrode before and after various discharge/charge cycles at 500 mA/g.

Figure 4A displays the typical CV profiles of ZnFe_2_O_4_/α-Fe_2_O_3_ nanocomposites electrode for the first four cycles between 0.01 and 3.0 V at a scan rate of 0.1 mV/s. During the first cycle, two intense reduction peaks can be observed at about 0.70 and 0.81 V, which can be associated with the inital reduction of ZnFe_2_O_4_ to metallic Fe^0^/Zn^0^ and the complete reduction of α-Fe_2_O_3_ to metallic Fe^0^, as well as the formation of amorphous Li_2_O matrix and solid electrolyte interface (SEI) film (Zhang Y. M. et al., 2017); the broad oxidation peak at about 1.63 V can be attributed to the oxidation of the metallic Zn^0^ and Fe^0^ to to Zn^2+^ and Fe^3+^, respectively (Yao et al., 2018c). In the subsequent cycles, the two reduction peaks merges together and shifts to 0.97 V, which can be due to the drastic lithium-driven structural and/or textural modifications on the electrode during the first lithiation process; the oxidation peak slightly shifts to 1.69 V, which is can be ascribed to structure rearrangement after the first lithiation/delithiation process (Zhang Y. M. et al., 2017; Zhou et al., 2017). The well-overlapped CV profiles of the third and the fourth cycle suggest the good electrochemical reaction reversibility of the nanocomposites electrode. Figure 4B gives the cycling performance of the ZnFe_2_O_4_/α-Fe_2_O_3_ nanocomposites electrode at a current density of 500 mA g^−1^. The initial discharge and charge capacities are 1,522 and 1,027 mAh g^−1^, with a coulombic efficiency of 67.5%. This large irreversible capacity loss during the first cycles is mainly caused by the irreversible reaction and formation of the SEI layer on the surface of electroactive materials (Reddy et al., 2013). The electrode exhibits a rapid capacity decay in the first 20 cycles and relatively slow capacity decay from the 20 to 80th cycle, which can be ascribed to the mechanical degradation and unstable SEI formation upon discharge/charge cycling (Sun et al., 2014). After 80 cycles, the discharge capacity increases gradually and gets 1,206 mAh g^−1^ in the 400th cycle. The rise of the discharge capacity may result from the synergistic combination of the refinement of nanoparticles and the formation of organic polymeric/gel-like layer by electrolyte decomposition with the increase of the number of cycles (Hassan et al., 2011; Rai et al., 2014; Wang M. Y. et al., 2017). Figure 4C presents the typical discharge/charge curves of the nanocomposites electrode in different cycles at a current density of 500 mA g^−1^. After the first discharge/charge cycle, the long discharge plateau change into a slope. The change of the voltage plateaus and the large capacity loss after the first cycle match well with the CV profiles (Figure 4A). Moreover, the voltage hysteresis between discharge and charge profiles enlarges in the first 100 cycles, and then reduces gradually, implying that the electrode state and polarization degree vary with discharge/charge cycles. Figure 4D displays the Nyquist plots of the nanocomposites electrodes recorded before cycling and after selected discharge/charge cycles. Each plot consists of one depressed semicircle or two semicircles in the high to moderate frequency region and an inclined line in the low frequency region. The depressed semicircles can be assigned to the resistance of Li^+^ migrating through the SEI film and the charge-transfer resistance at electrode/electrolyte interface (denoted as *R*_sf+ct_), and the inclined line represents the Warburg impedance related to Li^+^ diffusion process into the bulk of the electrode (Wang et al., 2015; Li et al., 2016; Kong et al., 2017; Lee et al., 2017). Before cycling, the Warburg straight line is almost vertical to the real-axis (capacitive behavior), suggesting that there is almost no detectable Li^+^ intercalation in the electrode for fresh cell. With the number of cycles increasing from 1 to 100, the Warburg straight line gradually decreases to an angle of ~45° to the real-axis, showing the characteristic of Li^+^ diffusion in the electrode; however, further increasing the number of cycles from 100 to 400, the Warburg straight line gets more and more steep, presenting the characteristic of pseudocapacitive behavior, which may derive from the reversible formation of organic polymeric/gel-like layer (Laruelle et al., 2002). The Nyquist plots were fitted by the equivalent circuits shown as inset in Figure 4E. In the circuits, the *R*_e_, *R*_sf_, and *R*_ct_ are electrolyte resistance, SEI layer resistance, and charge transfer resistance, respectively. *C*PE1 and *CPE*2 are the corresponding capacitances of *R*_sf_ and *R*_ct_. *W* is the Warburg impedance. The calculated *R*_sf+ct_ values of the electrodes before and after different discharge/charge circles is presented in Figure 4E. Before cycling, the *R*_sf+ct_ value of the electrode is 40 Ω. During the initial 100 cycles, the *R*_sf+ct_ value increases from 51 to 195 Ω; further increasing the number of cycles increase from 100 cycles to 300 and 400 cycles, the *R*_sf+ct_ value decreases from 195 to 80 and 77 Ω, respectively. The EIS results reveals that the decrease in capacity during the initial 80 cycles can be due to the slow Li^+^ diffusion process and large resistances from SEI film and charge-transfer process (high polarization); the increasing capacity in the following cycles from 80 to 400 cycles ca be explained by the enhanced Li^+^ diffusion process and reduced resistances from SEI film and charge-transfer process (low polarization).

**Figure 5:**
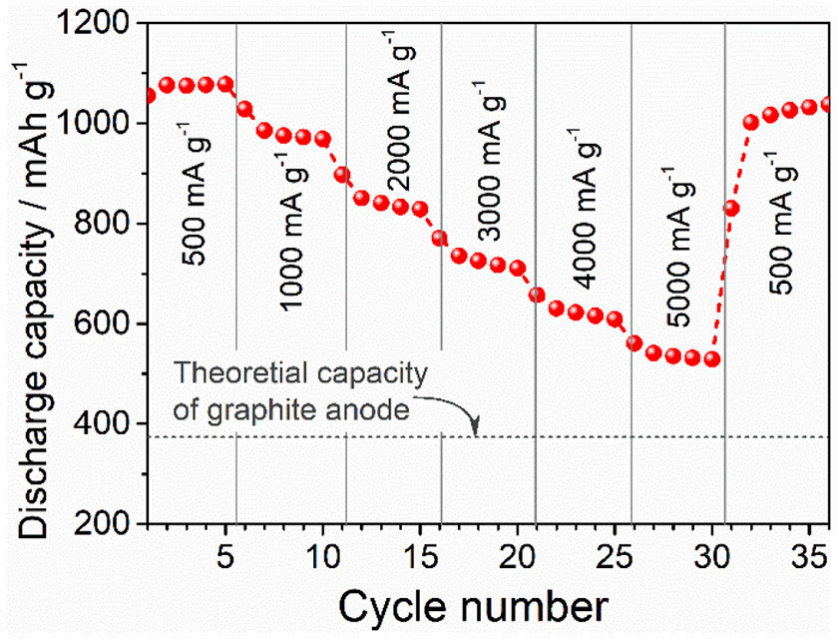
Rate performance of the ZnFe_2_O_4_/α-Fe_2_O_3_ nanocomposites electrode at various current densities after 250 cycles at a constant density of 500 mA g^−1^.

The rate performance of the of ZnFe_2_O_4_/α-Fe_2_O_3_ nanocomposites electrode was tested after the electrode is activated at 500 mA g^−1^ for 250 cycles, and the result are shown in Figure 5. With the increase of current density, the discharge capacity decreases gradually. Even at the very high current density of 5,000 mA g^−1^, the specific capacity (535 mAh g^−1^) still obviously higher than the theoretical capacity (372 mAh g^−1^) of graphite. When the current density reverses back to 500 mA g^−1^, the discharge capacity almost recovers to the original values, implying the good capacity retention performance of the electrode.

**Figure 6:**
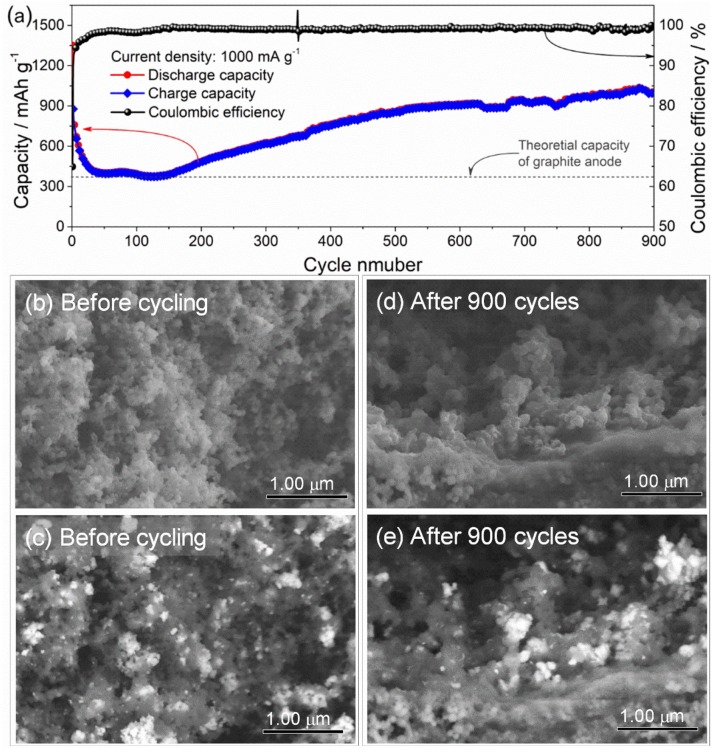
**(a)** Long-term cycling performance of the ZnFe_2_O_4_/α-Fe_2_O_3_ nanocomposites electrode at a current density of 1000 mA g-1. SEM images of the ZnFe_2_O_4_/α-Fe_2_O_3_ nanocomposites electrode **(b,c)** before cycling and **(d,e)** after 900 cycles. Figures **(c,e)** are back scattering images of Figures **(b,d)**, respectively.

Figure 6a gives long-term cycling performance of the ZnFe_2_O_4_/α-Fe_2_O_3_ nanocomposites electrode at current density of 1,000 mA g^−1^ for 900 cycles. It can be seen that the electrode exhibits excellent long-term cycling performance with a high capacity of about 1,000 mAh g^−1^ after 900 cycles at the constant current density of 1,000 mA g^−1^. Compared with previous work, the as-prepared ZnFe_2_O_4_/α-Fe_2_O_3_ nanocomposites deliver better electrochemical performance in terms of reversible discharge capacities and cycling stability (Table 2). Figures 6b–e presents the SEM images of the ZnFe_2_O_4_/α-Fe_2_O_3_ nanocomposites electrode before and after 900 cycles. To identify ZnFe_2_O_4_/α-Fe_2_O_3_ nanocomposites more clearly in the electrode, backscattered electron images are also provided. It can be seen that the as-prepared electrode shows the presence of ZnFe_2_O_4_/α-Fe_2_O_3_ nanocomposites constructed by interconnected primary nanocrystals before cycling (Figures 6b,c). After 900 cycles, the electrodes almost maintains the original morphology (Figures 6b,c); the ZnFe_2_O_4_/α-Fe_2_O_3_ nanocomposites can be clearly observed in the electrode and the nanocrystals are well interconnected with each other, implying the ZnFe_2_O_4_/α-Fe_2_O_3_ nanocomposites are flexible to alleviate volume expansion upon repeated discharge/charge cycles.

**Table 2:** Comparison of lithium storage performance of ZnFe_2_O_4_-based nanomaterials from previous work and this work.

**Samples**	**Current density (mA g^−1^)**	**Discharge capacity (mAh g^−1^)**	**References**
ZnFe_2_O_4_/carbon nanocomposites	200	1,090 after 400 cycles	Jiang et al., 2016
Mesoporous ZnFe_2_O_4_/graphene composites	1,000	870 after 100 cycles	Yao et al., 2014c
Nano sized ZnFe_2_O_4_/flake graphene composites	100	730 after 100 cycles	Yao et al., 2014a
Mesoporous ZnFe_2_O_4_/C composite microspheres	50	1,100 after 100 cycles	Yao et al., 2014b
ZnFe_2_O_4_/carbon nanodiscs	100	965 after 100 cycles	Jin et al., 2015
ZnFe_2_O_4_/γ-Fe_2_O_3_ nanoparticles	500	360 after 300 cycles	Bourrioux et al., 2017
ZnFe_2_O_4_ nanospheres/graphene composites	1,000	704 after 50 cycles	Shi et al., 2015
Nitrogen-doped carbon coated ZnFe_2_O_4_	1,000	700 after 1,000 cycles	Yue et al., 2015
ZnFe_2_O_4_/C hollow spheres	65	841 after 30 cycles	Deng et al., 2011
ZnFe_2_O_4_ nano-octahedrons	1,000	730 after 300 cycles	Xing et al., 2012
ZnFe_2_O_4_/α-Fe_2_O_3_	1,000	1,000 after 900 cycles	This work

**Figure 7:**
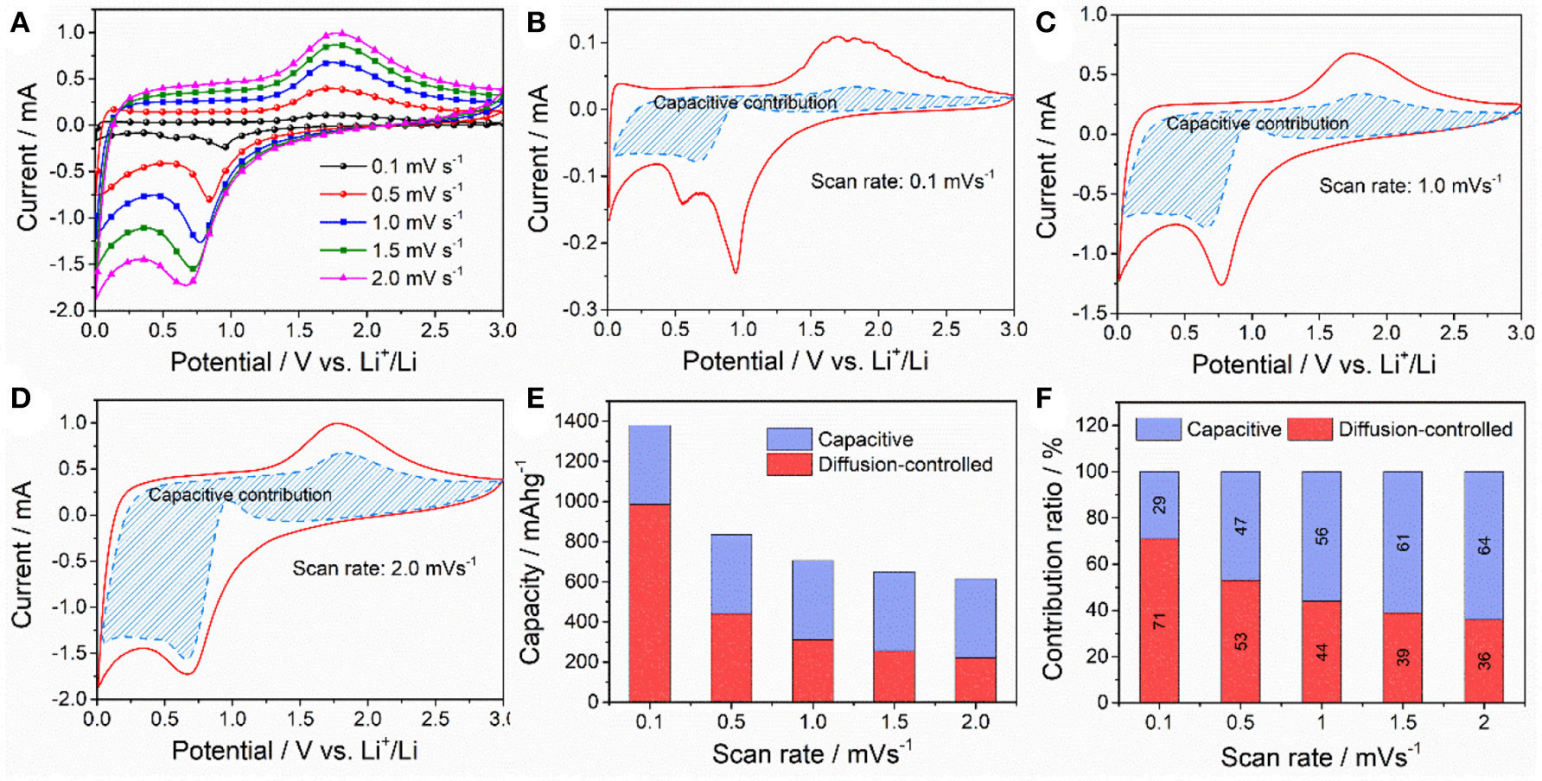
**(A)** CV curves of the ZnFe_2_O_4_/α-Fe_2_O_3_ nanocomposites electrode at various scan rates. **(B–D)** Separation of contributions from capacitance at different the scan rates of 0.1, 1.0, and 2.0 mV s^−1^, respectively (the blue shaded portions in the CV curves correspond to the capacitance). **(E)** Corresponding comparison of the total stored charge at different scan rates. **(F)** The percentages of pseudocapacitive contributions at different scan rates.

To better understand the superior lithium storage performance of the ZnFe_2_O_4_/α-Fe_2_O_3_ nanocomposites, we analyze the charge storage mechanism by the sweep voltammetry method proposed by Dunn et al. (Qu et al., 2017). In this method, the contributions from capacitive effect and diffusion-controlled Li^+^ process can be quantified by the following equations:

(1) $$\begin{array}{l} i\left(V\right)=k_{1}v+k_{2}v^{1/2} \end{array}$$

(2) $$\begin{array}{l} i\left(V\right)/v^{1/2}=k_{1}v^{2}+k_{2} \end{array}$$

where I(V) and ν represent the total current response at a given potential V and scan rate for the CV measurements; *k*_1_ν and *k*_2_ν^1/2^ represent the current due to surface capacitive effects and current due to diffusion-controlled reaction process, respectively. By determining *k*_1_ and *k*_2_, the currents arising from capacitive effect and diffusion-controlled Li^+^ process can be distinguished. Figure 7A gives the series of CV curves of ZnFe_2_O_4_/α-Fe_2_O_3_ nanocomposites electrode recorded at different scan rate. Figures 7B–D illustrate the typical voltage profiles for the capacitive current (blue shaded region) in comparison with the total current obtained at the scan rate of 0.1, 1.0, and 2.0 mV s^−1^, respectively. Obviously, capacitive charge storage contributes a significant proportion to the total capacity, in particular in the low potential region (0.8–0.01 V) during delithiation process. With the increase of scan rate, the portion from capacitive capacity increases dramatically. As shown in Figure 7F, the capacitive capacity makes up about 29% of the total capacity at the scan rate of 0.1 mV s^−1^, whereas this value increases to 56% and 64% at the scan rate of 1 and 2 mV s^−1^, respectively. Similar results have also been reported for the nano-sized NiO and Ni(OH)_2_ anode materials (Li Y. W. et al., 2017b; Zheng Y. Y. et al., 2018). This significant surface or near surface charge storage due to capacitive behavior benefits the high rate capability and cycling stability of electrode active materials (Rauda et al., 2013; Augustyn et al., 2014; Li Y. W. et al., 2017a).

The superior lithium storage performance of the ZnFe_2_O_4_/α-Fe_2_O_3_ nanocomposites can be ascribed to following several aspects: (1) the primary nanocrystals facilitate the transport of both Li^+^ and electrons because of the short diffusion distance, which enhances the kinetic performance; (2) the numerous void spaces among the interconnected primary nanoparticles and among the nanoparticles can accommodate the strain induced by the volume change during discharge/charge cycles, and therefore improve the cycling performance; (3) the unique ZnFe_2_O_4_/α-Fe_2_O_3_ heterojunctions provides an enhanced inner electric field at the interface between ZnFe_2_O_4_ and α-Fe_2_O_3_ nanocrystals, which may efficiently accelerate the charge-transfer kinetics during electrochemical reactions and boost the rate capability; (4) the significant pseudocapacitive behavior during discharge/charge process is also an important reason for the outstanding high rate capability and long-term cycling stability.

## Conclusions

Hybrid ZnFe_2_O_4_/α-Fe_2_O_3_ nanocomposites have been successfully fabricated with the leaching liquor of jarosite residue as raw material by a facile chemical coprecipitation method followed by heat treatment in air. The ZnFe_2_O_4_/α-Fe_2_O_3_ nanocomposites are composed of interconnected ZnFe_2_O_4_ and α-Fe_2_O_3_ nanocrystals with sizes in the range of 20–40 nm. Due to the unique heterojunction nanostructure, the ZnFe_2_O_4_/α-Fe_2_O_3_ nanocomposites exhibits high lithium storage activity, superior cyclic stability, and good high rate capability when evaluated as anode materials for lithium-ion batteries. The reversible capacity of 1,000 mAh g^−1^ is achieved over 900 cycles at the constant current density of 1,000 mA g^−1^; even at the high current density of 5,000 mA g^−1^, the specific discharge capacity of 535 mAh g^−1^ can be obtained, which is still significantly higher than the theoretical capacity (372 mAh g^−1^) of graphite. Charge storage mechanism analysis demonstrates that surface pseudocapacitive lithium storage has a significant contribution to the total stored charge of the ZnFe_2_O_4_/α-Fe_2_O_3_ nanocomposites, which accounts for the enhanced lithium storage performance during cycling. This work provides a facile, efficient, and low-cost method for the synthesis of high-performance Fe-based oxides anode materials by utilizing the leaching liquor of jarosite residue as raw material, which can make use of the industrial jarosite residue as resource, reduce the environment pollution, create high value-added products, and will achieve both good social and economic benefits.

## Author contributions

JinhY and YL conceived the idea. JingY prepared all materials and performed electrochemical characterizations. YH conducted SEM experiments. YL, JingY, JinhY, JX, and SX analyzed the data. JinhY and JingY wrote the manuscript. YL and JX commented on it. JinhY supervised the implementation of the project.

### Conflict of interest statement

The authors declare that the research was conducted in the absence of any commercial or financial relationships that could be construed as a potential conflict of interest.
